# Exploring Neuro-Physiological Correlates of Drivers' Mental Fatigue Caused by Sleep Deprivation Using Simultaneous EEG, ECG, and fNIRS Data

**DOI:** 10.3389/fnhum.2016.00219

**Published:** 2016-05-13

**Authors:** Sangtae Ahn, Thien Nguyen, Hyojung Jang, Jae G. Kim, Sung C. Jun

**Affiliations:** ^1^School of Electrical Engineering and Computer Science, Gwangju Institute of Science and TechnologyGwangju, South Korea; ^2^Department of Biomedical Science and Engineering, Gwangju Institute of Science and TechnologyGwangju, South Korea

**Keywords:** EEG/ECG/EOG/fNIRS, neuro-physiological correlates, drivers' mental fatigue, sleep deprivation, simulated driving, multimodal integration, driving condition level

## Abstract

Investigations of the neuro-physiological correlates of mental loads, or states, have attracted significant attention recently, as it is particularly important to evaluate mental fatigue in drivers operating a motor vehicle. In this research, we collected multimodal EEG/ECG/EOG and fNIRS data simultaneously to develop algorithms to explore neuro-physiological correlates of drivers' mental states. Each subject performed simulated driving under two different conditions (well-rested and sleep-deprived) on different days. During the experiment, we used 68 electrodes for EEG/ECG/EOG and 8 channels for fNIRS recordings. We extracted the prominent features of each modality to distinguish between the well-rested and sleep-deprived conditions, and all multimodal features, except EOG, were combined to quantify mental fatigue during driving. Finally, a novel driving condition level (DCL) was proposed that distinguished clearly between the features of well-rested and sleep-deprived conditions. This proposed DCL measure may be applicable to real-time monitoring of the mental states of vehicle drivers. Further, the combination of methods based on each classifier yielded substantial improvements in the classification accuracy between these two conditions.

## Introduction

Neuroergonomics is an emerging field that investigates human mental states and their workloads in order to improve the reliability of human performance, and ensure its stability in various environments (Parasuraman, 2003; Parasuraman and Rizzo, 2008). In neuroergonomics, both the fundamental principles of neuroscience and human factors are considered thoroughly, and neural behaviors have been investigated primarily when people are engaged in tasks in a work environment (Parasuraman and Wilson, 2008). Due to the implications for public safety, a major application of neuroergonomics is the assessment of driver fatigue. In general, driver fatigue is categorized as either mental or physical. Mental fatigue occurs because of gradual and cumulative mental effort (Grandjean, 1979) during driving, or sleep deprivation before driving (Durmer and Dinges, 2005). In contrast, physical fatigue represents reduced muscular strength and coordination. Physical fatigue may be countered by deliberate action; however, mental fatigue is difficult to resolve. Because of mental fatigue, drivers begin to doze involuntary, which often results in traffic accidents (Horne and Reyner, 1999; Connor et al., 2002; Herman et al., 2014).

One potential method that may be used to reduce traffic accidents is to measure inherent mental fatigue before or during driving, in order to predict a driver's mental condition and determine whether s/he can drive safely. Because driving requires complex cognitive processes and sustained concentration, predicting a driver's mental fatigue before or during driving could be effective in preventing traffic accidents. Thus, we attempted in this work to explore neuro-physiological correlates in two different conditions, one well-rested with a low risk of fatigue, and the other sleep-deprived with a high risk of fatigue.

Among many studies performed to evaluate drivers' fatigue in real-time, computer vision-based systems have been used widely. Bergasa et al. (2006) proposed a noninvasive system to monitor a driver's vigilance using several parameters, including percentage or duration of eye closure, blinking, and the frequency of nodding. By using a fuzzy classifier, the researchers then inferred the level of the drivers' fatigue. However, the reliability of the findings decreased when the drivers wore glasses or the surrounding brightness changed. To address these problems, D'Orazio et al. (2007) designed an experimental paradigm that incorporated conditions in which some subjects had different eye colors, wore glasses, and drove vehicles in light of varying intensities. Using the proposed visual framework, the authors obtained robust results. In addition, various visual cues that characterized eyelid, gaze, and head movements, as well as facial expressions were employed in a probabilistic model developed to predict fatigue (Ji et al., 2004) that yielded even more robust results. Recently, Wang et al. (2014) developed an online, closed-loop lapse detection system featuring a mobile wireless electroencephalograph (EEG), and were able to extract certain EEG signatures associated with fatigue.

To date, EEG has been found to be a promising indicator for investigations of driver fatigue (Lal and Craig, 2001). EEG data have shown that there is a significant increase in theta and delta activity, and a decrease in heart rate (HR) associated with fatigue (Lal and Craig, 2002). Further, in a subsequent study that considered three phases of fatigue (early, medium, and extreme), software was developed to monitor driving fatigue, and was validated with EEG data from 35 subjects engaged in a simulated driving task (Lal et al., 2003). Another study (Lin et al., 2005) estimated drowsiness and driver performance by correlating changes in log power spectra. To detect drowsiness, they constructed an individualized linear regression model to assess EEG dynamics continuously based on an independent component analysis. Because drowsiness is a crucial factor in driving, Lin and his colleagues investigated the effect of continuous arousing auditory feedback on sustained attention in a driving simulator (Lin et al., 2010). They found that spectral powers in alpha and theta bands were suppressed and lasted 30 s or longer after feedback. This finding was introduced to estimate classification accuracy; as a result, they achieved a classification accuracy of approximately 78% using the maximum likelihood classifier (Lin et al., 2013) and applied it to develop an online, closed-loop system for practical lapse detection in real environments (Wang et al., 2014).

Various other methods have been used to explore drivers' mental fatigue, such as a support vector machine (SVM) (Shen et al., 2008; Yeo et al., 2009), Bayesian network (Yang et al., 2010), wavelet analysis (Kar et al., 2010; Li and Chung, 2013), and others. In addition to EEG studies, electrocardiography (ECG) and electrooculography (EOG) have been used to determine neuro-physiological correlates of drivers' mental fatigue. One study (Patel et al., 2011) used neural network analysis and demonstrated that the variability in drivers' HRs differed significantly in alert and fatigued states. They investigated the power spectral density behaviors between the two states during long-term driving and reported that the neural network was 90% accurate in classifying mental state.

Eyelid-related features from EOG data also have been reported to be possible candidates to detect whether or not a driver is drowsy (Hu and Zheng, 2009). In this report, they used vertical and horizontal EOG channels to extract and validate eye blinks according to eyelid movement parameters, such as blink duration, speed, and amplitude. Three conditions (alert, sleepy, and very sleepy) were classified with high reliability using SVM. Simultaneous recording of EEG/ECG (Zhao et al., 2012) and the combination of multimodal features from EEG, EOG, and ECG data (Khushaba et al., 2011) demonstrated significant differences during long-term driving. In this study, the researchers developed an efficient, fuzzy mutual information-based wavelet packet transformation that combined EEG, EOG, and ECG features to detect drivers' drowsiness; this technique yielded a classification accuracy greater than 90%.

An emerging portable and noninvasive brain functional imaging technique, functional near infra-red spectroscopy (fNIRS), has been introduced to monitor cognitive workload or fatigue in simulated environments (Ayaz et al., 2012). fNIRS data from the prefrontal cortex were collected during a complex air-traffic control task that required the subjects to prevent collisions between aircraft in their sectors. As the number of aircraft in their sector increased, a concomitant increase in prefrontal cortex activation was observed, which suggests that fNIRS provides a sensitive index of cognitive workload. fNIRS also demonstrated changes in prefrontal activation during skill acquisition in both basic working memory tasks (McKendrick et al., 2014) and more complex piloting tasks (Harrison et al., 2014; Gateau et al., 2015).

A portable fNIRS device was developed for use in mobile neuroimaging of the prefrontal cortex (Ayaz et al., 2013). In a driving environment, Li et al. (2009) observed changes in cerebral oxygenation during prolonged simulated driving. Forty healthy subjects were divided randomly into two groups (driving vs. non-driving), and the driving group performed a simulated 3 h driving task. The authors found a relative increase in frontal cortex oxygenation in the driving group by comparison to the non-driving group, and oxygenation decreased gradually after the driving task. Considering real driving situations, Yoshino et al. (2013) investigated the changes in cerebral oxygen exchange during actual driving on an expressway. An fNIRS signal was recorded in the subjects' parietal and prefrontal cortices using an fNIRS device mounted in the vehicle. They found that the areas activated varied depending on the driving task, such as parking, acceleration, driving at constant speed, deceleration, and U-turns. Thus, the use of fNIRS may be an effective approach to evaluate brain activity in various driving environments.

Recently, hybrid approaches that combine two different modalities (Pfurtscheller et al., 2010) to improve performance and reduce classification error have been reported as promising for future brain-computer interfaces (BCI). One example of a hybrid BCI that incorporates both EEG electrical activity and fNIRS hemodynamic changes yielded improved classification performance in sensorimotor rhythm-based BCI systems (Fazli et al., 2012). The researchers calculated classification accuracies in the movements executed and motor imagery by estimating a meta-classifier. After the estimation of both classifiers (EEG and fNIRS), the combination of outputs of each classifier resulted in improved classification accuracy. Khan et al. (2014) decoded four movement directions (left, right, forward, and backward) using the mixed features of EEG and fNIRS, in which EEG features were used to classify left/right, and fNIRS features were used to classify forward/backward. In addition, hybrid BCI may be used as a brain switch that determines whether a certain task is active. Koo et al. (2015) employed a novel experimental paradigm to detect the occurrence of motor imagery in fNIRS data. Threshold-based detection with a feature value of the fNIRS data determined whether or not the action of a motor imagery task was attempted. The combination of EEG and fNIRS is also applicable to language studies (Wallois et al., 2012) and cortical current estimation (Morioka et al., 2014). Hybrid BCIs may provide a good opportunity to increase BCI performance by offering the synergistic effects of multimodal brain imaging techniques.

In this work, we recorded multimodal EEG/ECG/EOG and fNIRS data simultaneously in a driving simulator and combined their features to distinguish drivers with high- and low-risks of fatigue using neuro-physiological correlates and a classification method. Hemodynamic changes in the prefrontal cortex (Li et al., 2009; Ayaz et al., 2012, 2013; Yoshino et al., 2013; Harrison et al., 2014; McKendrick et al., 2014; Gateau et al., 2015) have been used to neuro-physiological correlates, and these activities were reported to play an important role in neuroergonomics, such as mental workload (Mandrick et al., 2013a), cognitive operation (Mandrick et al., 2013b), and emotional function (Doi et al., 2013). Furthermore, it is clear that EEG, ECG, and EOG are also promising indicators that may be used to investigate the neuro-physiological correlates of drivers' mental fatigue (Lal and Craig, 2001). Therefore, combining this hybrid system with prefrontal fNIRS may be a far more informative measure for identifying neuro-physiological correlates under varying driving conditions. To the best of our knowledge, this multimodal approach has been tested rarely to explore neuro-physiological correlates of drivers' mental fatigue.

Thus, the goal of this study was to determine modality-specific features of EEG, EOG, ECG, and fNIRS. These features were then used to distinguish between well-rested and sleep-deprived conditions, and resulted in a classifier that showed whether or not a driver was in an alert mental state. The use of a reasonable combination of these multimodal features may improve classification accuracy and its quantification may yield a real-time strategy to monitor drivers' mental fatigue.

## Materials and methods

### Experimental procedure

**Figure 1:**
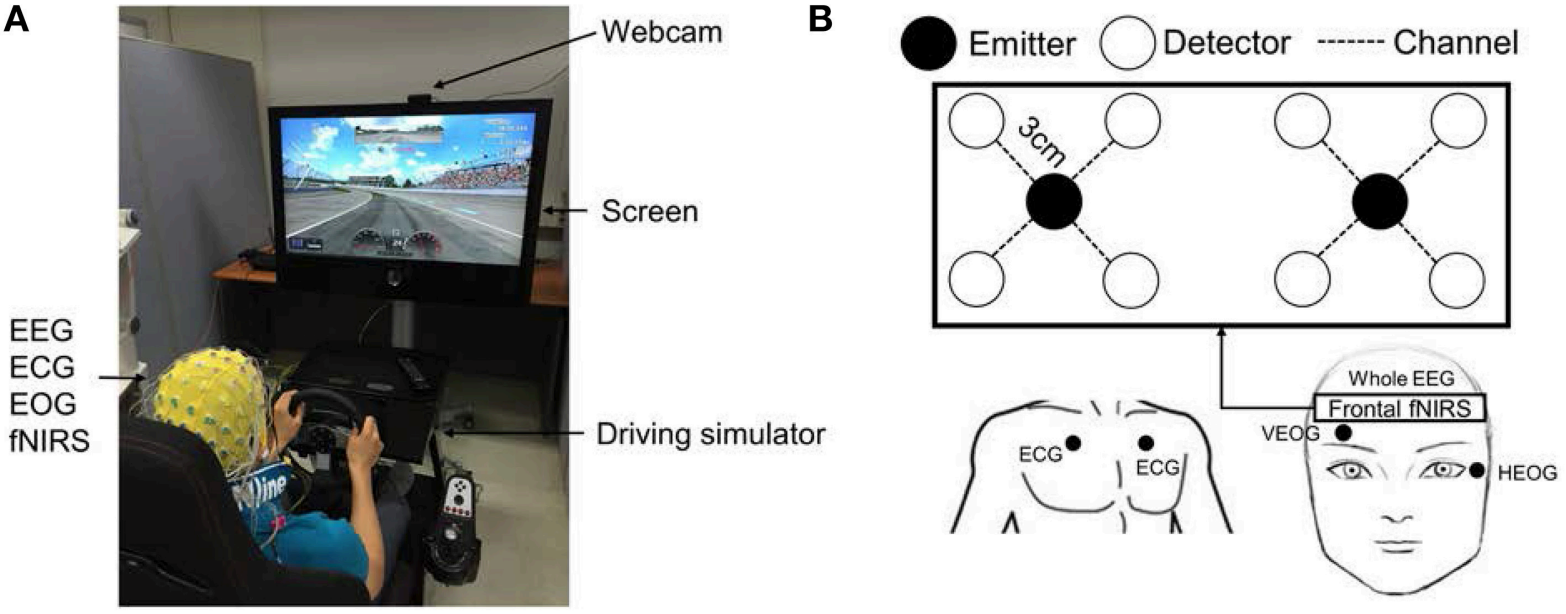
**Experimental setup for simulated driving**. **(A)** Driving simulation environment with EEG/ECG/EOG/fNIRS measurements. **(B)** fNIRS setup in the prefrontal cortex. Two emitters and eight detectors (eight channels) were attached to the forehead. The distance between emitter and detector was 3 cm.

Eleven healthy subjects (10 males, 1 female, aged 26.6 ± 1.4, range = 24–28) who had valid driver's licenses participated in a custom-built virtual driving simulation task, as depicted in Figure 1A. The subjects practiced repeatedly until they were familiar with the simulation system. The purposes of, and instructions for, the experiment were explained in advance, and all of the subjects signed an informed consent. Subjects received approximately $10 per h as compensation for their participation. Each subject performed simulated driving under two conditions (well-rested and sleep-deprived) on different days. Under the well-rested condition, subjects were instructed to sleep at least 7 h before the experiment, as sleeping seven or more hours is known to maintain healthy mental alertness (Kripke et al., 2002). In the sleep-deprived condition, the subjects were instructed to stay up all night in order to produce mental fatigue.

Driving tests in both conditions were performed before 9 a.m. In this experiment, we assumed that subjects would be significantly mentally fatigued after one night of sleep deprivation. To determine the degree of fatigue produced by sleep deprivation, a subjective questionnaire was administered to the subjects before the experiment to score their levels of fatigue, and the scores demonstrated clearly that the sleep-deprived subjects were substantially more fatigued than were the well-rested subjects. The subjects sat in a comfortable driver's seat and drove on an oval track for a minimum of 30 min. The maximum driving speed was set at 100 km/h in both conditions. The steering wheel vibrated whenever the vehicle collided with a crash barrier in order to prevent the drivers from falling asleep completely. A high-definition webcam (Logitech HD Pro C920) was used to record each subject's behavior in real-time. This experiment was approved by the Institutional Review Board at the Gwangju Institute of Science and Technology (20150615-HR-18-02-06).

### Data recording of EEG/ECG/EOG and fNIRS

Sixty-four EEG electrodes were attached to the drivers' scalps according to the 10–20 international position system. Horizontal and vertical EOGs were used and two ECG electrodes were attached to the left/right chest (Biosemi ActiveTwo System). These data were collected at a 512 Hz sampling rate using BCI2000 software (Schalk et al., 2004). Biosemi ActiView software monitored the stability and reliability of the EEG signal. After the experiment, bad channels that contained abnormal noise were identified by visual inspection and excluded from the analysis.

A custom-built fNIRS system (continuous wave, 10 Hz sampling rate) was used to record hemodynamic changes in the brain. This was an updated version of one described in a previous work (Kim et al., 2015). The system consists of probe and control circuits. The probe includes 2 LEDs (emitters) and 8 photodetectors (detectors). The LEDs emit near infrared (NIR) light at two wavelengths (735 and 850 nm). The emitter and four surrounding detectors were separated by 3 cm, as Homma et al. (1996) suggested that in soft tissues, NIR is able to attain a penetration depth equal to half of the emitter-detector separation. Therefore, with a 3.0 cm emitter-detector separation, our system should have been able to collect brain activity at a depth of 1.5 cm below the scalp. An emitter-detector pair form one fNIRS channel that measures hemodynamic changes midway between the emitter and the detector. Given a suitable geometric arrangement, many detectors may receive light from one emitter. This enabled us to design an 8-channel probe with 2 LEDs and 8 photodetectors. The 8-channel probe was attached to the prefrontal region to investigate the subjects' mental state, as illustrated in Figure 1B (Li et al., 2009; Sato et al., 2013). The control circuit receives a signal from the probe, amplifies it, and sends it to the computer via serial communication. Matlab-based software was programmed to record, process, and display the hemodynamic signals. Interference between EEG/EOG/ECG electrodes and fNIRS emitters has been observed and is believed to result from light leakage from the emitters, which may cause deterioration in the quality of electrical data (Koo et al., 2015). This interference was removed by blocking light leakage from the emitters and applying a simple pre-processing technique. Two desktop computers were used to record the EEG/ECG/EOG and fNIRS data simultaneously. Triggers for start and end times were sent to the BCI2000 software to synchronize the multimodal EEG/ECG/EOG and fNIRS data. The computer that recorded EEG/ECG/EOG data sent a start trigger, at which time the second computer began to record fNIRS data. The end time of the experiment was marked in the same way.

### Data analysis

#### Feature extraction and classification from EEG

After the experiment, the data collected were inspected visually and bad channels were rejected. The logistic infomax independent component analysis (Bell and Sejnowski, 1995) was used to remove EOG artifacts and the data were then band-pass filtered from 1 to 50 Hz. We analyzed data from the first 30 min only after the drivers began the task, because fatigue levels between well-rested and sleep-deprived subjects were likely to be quite different during the initial minutes of driving. From the real-time webcam video monitoring data, we observed that even some well-rested subjects became drowsy and quite bored after that length of time.

EEG data (30 min) were divided into 10 s (a trial) to yield a total of 180 trials for each driving condition. A power spectral density was computed for each trial using the EEGLAB library (Delorme and Makeig, 2004), and a relative power level (RPL) was computed in order to reduce session/subject variability (Ahn et al., 2013a,b, 2014; Cho et al., 2015). To calculate the RPL, we considered five spectral band ranges: delta (1–4 Hz), theta (4–8 Hz), alpha (8–13 Hz), beta (13–30 Hz), and gamma (30–50 Hz). Next, each band-power was normalized by the total power, defined as the sum over all band powers, after which we extracted the most informative RPL features between the two driving conditions. On the other hand, to discriminate between the well-rested and sleep-deprived conditions, pre-processed data (180 trials) from each driving condition (well-rested and sleep-deprived) were firstly divided into 2 groups (training and test) and according to time sequence; training and test groups were composed of 126 (70%) and 54 trials (30%), respectively. Then, to avoid temporal dependency between groups, last 6 trials (1 min) for each group were excluded; thus, for each of driving conditions, 120 and 48 trials for training and test were obtained, respectively. By this grouping, temporal dependency (adjacency) was included within groups, but was excluded between groups. This procedure was repeated 30 times by sliding temporal window of 1 min (6 trials) and then choosing training and test groups. Thereafter, each feature vector of the training and test data using RPL was fed into the classifier. The training group was used to construct a classifier based on Fisher's linear discriminant analysis (FLDA), and the test group was input to a constructed classifier in order to measure classification accuracy. A classifier was generated from the training data and the classification accuracy was estimated from the test data. Finally, 30 classification accuracies were estimated to obtain an average accuracy.

#### Feature extraction and classification from ECG and EOG

The HRs of each subject were extracted using two ECG channels (left/right chest). During pre-processing, ECG data were band-pass filtered from 0.1 to 30 Hz and were detrended to remove the baseline shift. After detrending, a QRS-complex was observed to be the most prominent repeating peak in the ECG signal. The QRS-wave is used commonly to determine subjects' HRs or predict abnormalities in cardiac function. Specifically, the emergence of an R-peak indicated a subject's HR clearly and was extracted easily by adjusting a deterministic threshold of the ECG magnitude. Next, the number of R-peaks per minute was counted and used to determine HR per minute. HRs from the two ECG channels on the left and right chest were calculated for the entire 30 min and averaged to reduce possible detection error and bias. To classify mental state from the ECG data, we adopted the extraction of RR-peak interval features (de Chazal et al., 2004). After detection of the R-peak in each 10-s trial, the intervals between one R-peak and the next were averaged, and the procedure was repeated for all trials. In this way, 180 R-peak intervals were estimated as a feature set. The EOG signal was used to extract the rate of eye blinking in each 1-min trial, which has been reported to be associated well with a human's mental state (Schleicher et al., 2008): when the eye blinks, a clear, sharp wave is observed. After baseline drift removal was applied, a peak detection algorithm (Pettersson et al., 2013) was used with a given threshold of signal magnitude. Finally, the number of peaks per minute, which represented the eye-blinking rate, was used as the EOG feature.

#### Feature extraction and classification from fNIRS

We adopted the modified Beer-Lambert's law (mBLL) to retrieve relative concentration changes from the light intensities of the 8 detectors (Cope et al., 1988; Kocsis et al., 2006). The change in optical density at two wavelengths (735 and 850 nm) is related to changes in oxy-hemoglobin concentration (HbO) and deoxy-hemoglobin concentration (HbR). Data with abnormal noise were removed by visual inspection, and the remaining data were filtered with a 0.01 Hz high-pass filter to remove baseline drifts. Light intensities for 30 s after the initiation of the experiment were averaged and set as baseline intensities. HbO and HbR were estimated with the following equations:

(1) $$\begin{array}{r} △HbO=\frac{log\frac{I_{b}^{\lambda_{1}}}{I_{t}^{\lambda_{1}}}\varepsilon_{HbR}^{\lambda_{2}}-log\frac{I_{b}^{\lambda_{2}}}{I_{t}^{\lambda_{2}}}\varepsilon_{HbR}^{\lambda_{1}}}{d\cdot DPF\left[{\varepsilon_{HbO}^{\lambda_{1}}\varepsilon_{HbR}^{\lambda_{2}}-\varepsilon }_{HbO}^{\lambda_{2}}\varepsilon_{HbR}^{\lambda_{1}}\right]}, \end{array}$$

(2) $$\begin{array}{r} △HbR=\frac{log\frac{I_{b}^{\lambda_{2}}}{I_{t}^{\lambda_{2}}}\varepsilon_{HbO}^{\lambda_{1}}-log\frac{I_{b}^{\lambda_{1}}}{I_{t}^{\lambda_{1}}}\varepsilon_{HbO}^{\lambda_{2}}}{d\cdot DPF\left[{\varepsilon_{HbO}^{\lambda_{1}}\cdot \varepsilon_{HbR}^{\lambda_{2}}-\varepsilon }_{HbO}^{\lambda_{2}}\cdot \varepsilon_{HbR}^{\lambda_{1}}\right]}, \end{array}$$

where

$I_{b}^{\lambda }$ : *baseline intensity* (λ_1_ : 735 *nm* λ_2_ : 850 *nm*)

$I_{t}^{\lambda }$ : *transient intensity*

d: *emitter* − *detector separation*

$\varepsilon_{Hb}^{\lambda }$ : *extinction coefficient*

*DPF*:*differential path length factor*

In continuous wave fNIRS, the differential path length factor (DPF) is unknown. However, it is similar for both wavelengths and is included conventionally in the unit of hemodynamic changes as a scaling factor. Thus, HbO and HbR have the same unit of mM/DPF, and the extinction coefficients are specific for HbO and HbR at each wavelength. Matcher et al. (1995) measured extinction coefficients of hemoglobin at different wavelengths as follows:

at wavelength λ_1_= 735 nm,

$$\begin{array}{l} \varepsilon_{HbO}^{\lambda_{1}}=0.4646\text{m}{M^{-1}cm}^{-1}\text{ and }\varepsilon_{HbR}^{\lambda_{1}}=1.2959\text{m}{M^{-1}cm}^{-1}, \end{array}$$

at wavelength λ_2_= 850 nm,

$$\begin{array}{l} \varepsilon_{HbO}^{\lambda_{2}}=1.1596\text{m}{M^{-1}cm}^{-1}\text{ and }\varepsilon_{HbR}^{\lambda_{2}}=0.7861\text{m}{M^{-1}cm}^{-1}. \end{array}$$

Like EEG feature extraction, 10 s of data were defined as one trial, which yielded a total of 180 trials per condition. Next, relative concentration changes were estimated for each trial. To reduce the effects of fluctuations and noise, fNIRS data were smoothed using 10-s temporal windowing with a 50% overlap. Finally, the amplitudes of HbO and HbR were used as informative features for classification of the two driving conditions.

## Results

### Relative power level from EEG

**Figure 2:**
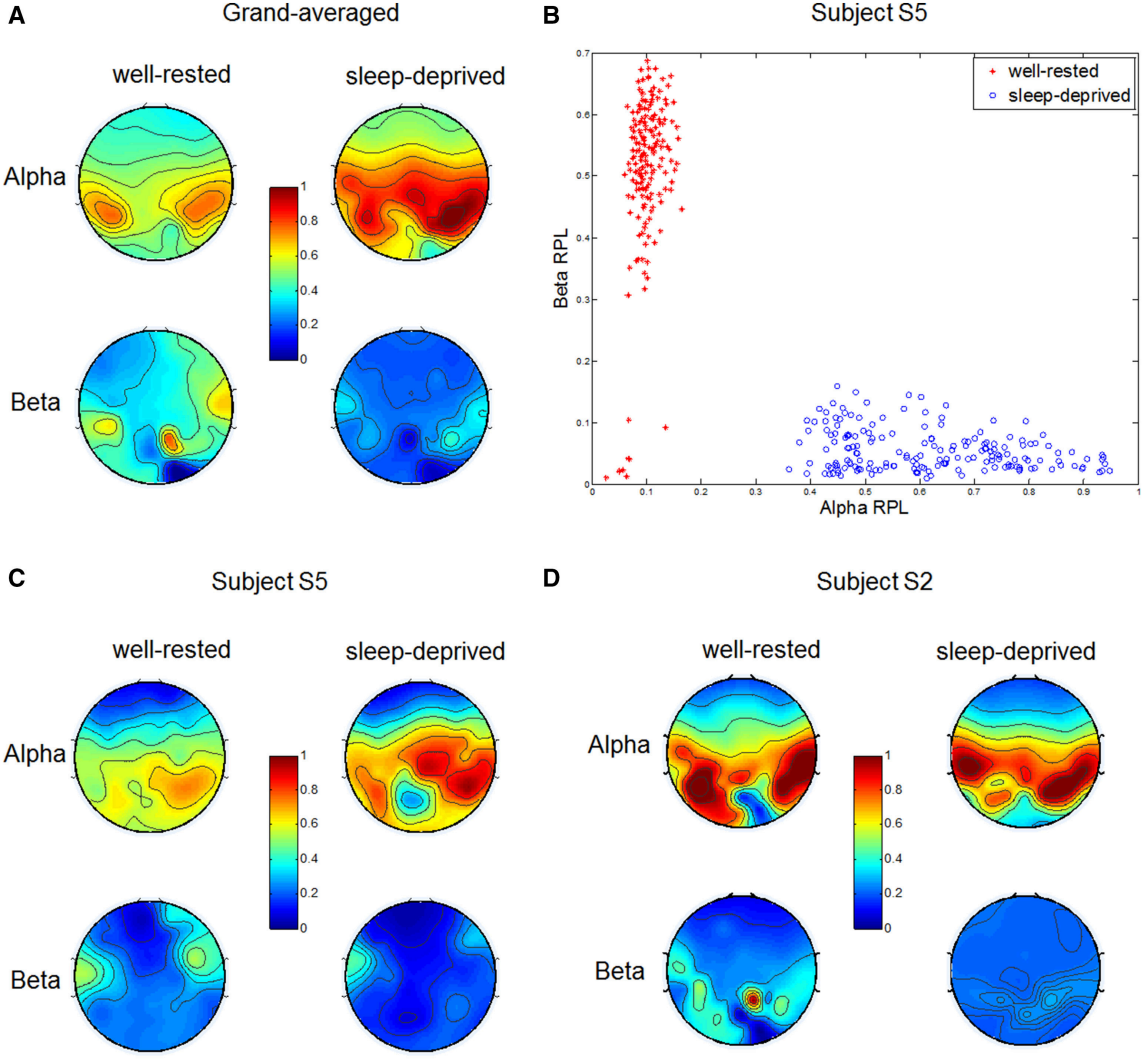
**Relative power levels from EEG in two different conditions**. **(A)** Grand-averaged alpha and beta RPLs in well-rested and sleep-deprived conditions. Alpha and beta RPLs differed significantly in the right centro-parietal and frontal regions, respectively. **(B)** Scatter plot of alpha (x-axis) and beta (y-axis) RPLs in two driving conditions (red asterisk: well-rested, blue circle: sleep-deprived) for subject S5. **(C,D)** Indicate alpha and beta RPLs in the well-rested and sleep-deprived conditions, in which subject S5 had the highest and S2 had the lowest classification accuracy, respectively.

We investigated RPL values over five spectral bands—delta, theta, alpha, beta, and gamma—and found that the RPL values for delta, theta, and gamma did not differ statistically between the two driving conditions. However, alpha and beta RPL values differed clearly in the two conditions, as shown in Figure 2A. Grand-averaged topographies were described for each condition, and alpha RPL in the sleep-deprived condition was activated to a greater degree in the right centro-parietal region. Such an increase in alpha power has been reported in the literature as a notable marker in driving (Lal and Craig, 2001; Simon et al., 2011). A decrease in beta RPL over the fronto-central region was observed in the sleep-deprived condition. This decrease in beta power may indicate a lack of arousal, which is consistent with the results of several studies (Tanaka et al., 2012; Zhao et al., 2012).

Figure 2B shows the distributions of alpha and beta RPLs from subject S5 in a two-dimensional Cartesian coordinate space. We note that most subjects showed similar physiological behaviors, and S5's results were chosen as representative because they yielded the highest classification accuracy in the EEG, as shown in **Table 3**. For the purposes of consistency and comparison, the other results (ECG, fNIRS) from subject S5 are also illustrated in the subsequent sections. Each RPL was averaged spatially over significant regions, such as the centro-parietal for alpha and the fronto-central for beta. Each dot represents corresponding alpha (x-coordinate) and beta (y-coordinate) RPLs for one trial in each condition (well-rested and sleep-deprived). In the well-rested condition, most RPL dots were distributed in the upper left area in R^2^ space, while they were distributed in the lower right area in the sleep-deprived condition. Thus, these features (centro-parietal alpha RPL and fronto-central beta RPL) in the dataset collected allowed us to achieve a discriminative classification between the two driving conditions quite well.

To investigate inter-subject variability (Ahn and Jun, 2015), we plotted RPL topographies in Figures 2C,D, respectively, for two subjects who achieved the highest (S5) and the lowest (S2) EEG classification accuracies (**Table 3**). As depicted in the figures, subject S5 showed a clear alpha RPL increase in the right centro-parietal region and beta RPL decrease in the fronto-central region in the sleep-deprived condition. In contrast, subject S2, who demonstrated the lowest classification accuracy, showed a slight alpha RPL increase and a beta RPL decrease in the sleep-deprived condition. Interestingly, this subject (S2) was likely to have been fatigued already, despite being in the well-rested condition before the experiment. Our investigation of this subject will be described in detail in the Discussion section.

### Time course of relative concentration changes from fNIRS

**Figure 3:**
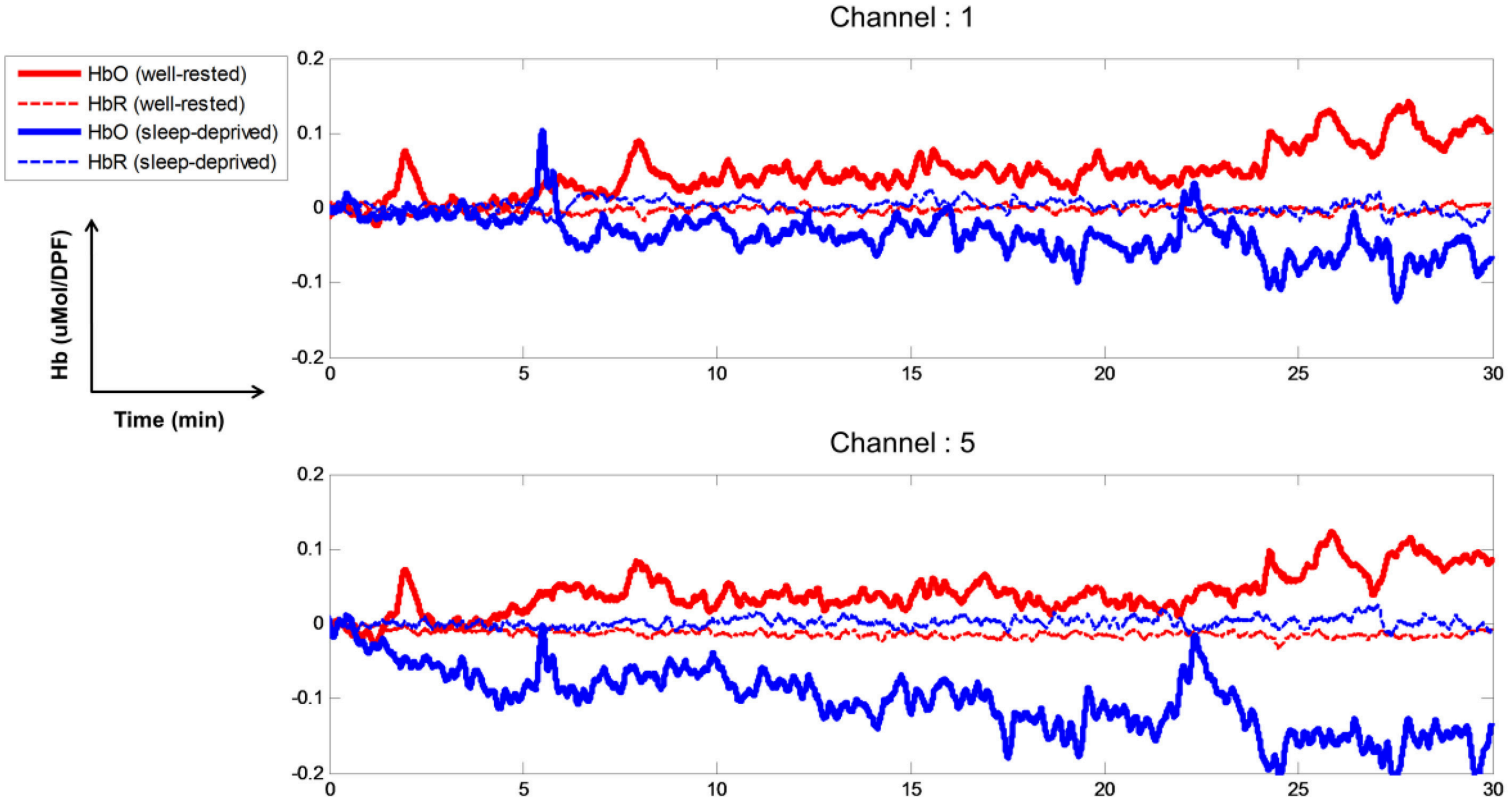
**Time course of relative concentration changes of HbO and HbR (channels 1 and 5)**. Solid, thick red and blue-colored lines indicate HbO for well-rested and sleep-deprived conditions, respectively. Dashed thin lines indicate HbR in the two conditions.

The time course of the relative concentration changes of HbO and HbR were estimated through mBLL. Figure 3 depicts the concentration changes at channels 1 and 5 for subject S5. Because all channels were attached to the prefrontal cortex, they all showed similar behaviors over time. Thus, for the purpose of illustration, we chose two representative channels (1 and 5). The concentration changes of HbO increased gradually over time in the well-rested condition and demonstrated the highest level at channels 1 and 5 between 20 and 30 min. Paying attention while driving a vehicle requires high oxygen consumption in the brain, which induces an increase in cerebral blood flow; this increase in cerebral oxygenation, as shown by an increase in HbO and decrease in HbR, indicates that the cerebral blood flow increased during driving under the well-rested condition. On the other hand, concentration changes of HbO decreased slightly (Channel 1) or remained stable (Channel 5) compared to the baseline, and HbR concentration maintained baseline values while driving under the sleep-deprived condition. Less oxygen may be consumed when mentally fatigued, and therefore, brain activity is likely to be suppressed, resulting in less blood flow to the brain.

### Reduced heart rate and eye blinking in the sleep-deprived condition

**Figure 4:**
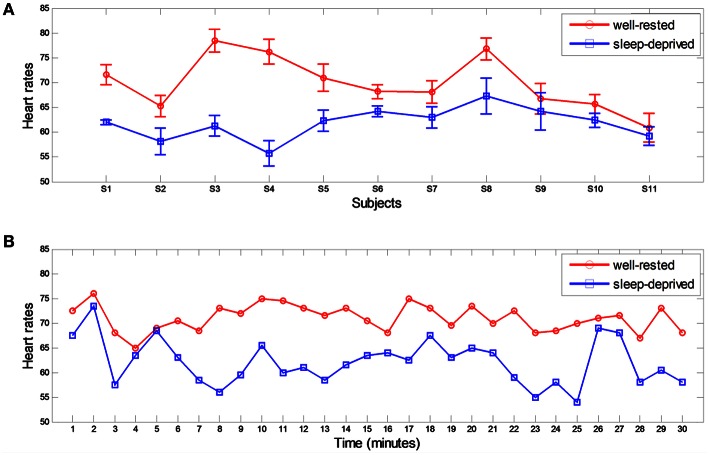
**Heart rates from ECG in two different conditions**. **(A)** Averaged heart rates for all subjects in well-rested and sleep-deprived conditions. **(B)** Subject 5's HRs over time. Each HR was estimated every minute.

The mean HRs of all subjects were calculated from ECG signals over the entire 30-min driving period (average of varied HRs), as tabulated in Table 1 and depicted in Figure 4A. As shown in the table and figure, HRs in the sleep-deprived condition were significantly lower than were those in the well-rested condition for all subjects (*p* < 0.01, Wilcoxon signed-rank test). Figure 4B shows the HR of subject S5 over time (from initiation to 30 min of driving). HRs in the well-rested condition were higher than those in the sleep-deprived condition; however, the difference in the HR between the two conditions decreased gradually as driving time increased and became quite small at the end of the driving task (~30 min). We deduced from this time variance in the HR that even a well-rested driver began to feel fatigued after some duration of driving and was considerably fatigued by the end of the task.

**Table 1:** **Averaged heart rates (HRs) in well-rested and sleep-deprived conditions**.

**Subject**	**S1**	**S2**	**S3**	**S4**	**S5**	**S6**	**S7**	**S8**	**S9**	**S10**	**S11**	**Mean**	***p*-value**
Well-rested	71.6 (2.0)	65.3 (2.2)	78.5 (2.3)	76.2 (2.5)	71 (2.8)	68.2 (1.4)	68.1 (2.3)	76.8 (2.2)	66.7 (3.1)	65.7 (1.9)	60.9 (2.9)	69.9 (3.3)	0.0009
Sleep-deprived	62 (0.5)	58.2 (2.7)	61.3 (2.1)	55.7 (2.6)	62.3 (2.2)	64.2 (1.1)	63 (2.2)	67.3 (3.7)	64.2 (3.8)	62.4 (1.4)	59.2 (1.9)	61.8 (3.5)	

*Values in parentheses indicate standard deviations for each HR. The Wilcoxon signed-rank test was performed*.

Because of the EOG signal, we expected that subjects in the sleep-deprived condition would demonstrate a relatively higher rate of eye blinking than those in the well-rested condition. Instead, we observed (not shown here) that some subjects showed higher rates of eye blinking in the well-rested than in the sleep-deprived condition, although the differences were not statistically significant. From the video data, we found that these subjects closed and opened their eyes frequently to overcome drowsiness, and this action on their part may have affected seriously the rate of eye blinking derived from the EOG data.

### Driving condition level and relative driving condition level

We attempted to demonstrate that it may be possible to evaluate neuro-physiological correlates of drivers' mental fatigue using the significant features found in EEG, ECG, and fNIRS data. To that end, we used the three factors extracted from multimodal signals in the previous sections: RPL (ratio of beta to alpha) from EEG, HbO from fNIRS, and the averaged HR from ECG. Each feature was normalized by scaling between 0 and 1 for equal contribution, as formulated in Equation (3). Each value of the EEG, ECG, and fNIRS was distributed well between those values. Significantly abnormal values—greater than 95% (mean ± 2^*^σ)—were considered outliers and were shrunk to their maximum or minimum values in the feature set. The summation of all three normalized factors was proposed as the driving condition level (DCL), as depicted in Equation (4). In addition, we estimated the relative difference in DCL between the well-rested and sleep-deprived conditions (rDCL), which represented the degree of the drivers' fatigue compared to that in the well-rested condition, as defined in Equation (5); a higher rDCL indicates greater fatigue.

(3) $$norm(x)=\frac{x-\text{min}(x)}{\max\left(x\right)\;-\text{min}(x)},$$

(4) $$\begin{array}{l} DCL\;=norm\text{​}\left(\frac{beta\;RPL}{alpha\;RPL}\right)+norm(HbO)\\ \text{ + }norm(HR),\\ \;(\text{0}\leq \text{n}orm\text{​}\left(\frac{beta\;RPL}{alpha\;RPL}\right)norm(HbO)norm(HR)\\ \;\leq \text{1},\text{ 0}\leq \text{DCL}\leq \text{3}) \end{array}$$

(5) $$rDCL\;(\%)=100-\frac{DCL_{sleep-deprived}}{DCL_{well-rested}}*100,$$

Using our proposed definition of DCL (Equation 4), DCL values were estimated in the two conditions for each subject. For all subjects, 30 min of multimodal data were used to estimate the values. DCL ranged from 0 to 3, with a smaller DCL indicating greater fatigue. These DCL values are tabulated in Table 2. Subject S8 showed the highest DCL value in the well-rested condition, while subject S4 showed the lowest DCL value in the sleep-deprived condition. We found that the two drivers' conditions (well-rested and sleep-deprived) differed significantly (*p* < 0.01, Wilcoxon signed rank test). In addition, rDCL, which represents the percentage of the fatigue in a drivers' mental condition, was introduced in this work. All rDCLs were consistently greater than 30%, except for those for two of 11 subjects (S7 and S9); thus, when rDCL is tuned more finely with more data, it may be used as a predictor of mental fatigue.

**Table 2:** **Driving condition level (DCL) in well-rested and sleep-deprived conditions**.

**Subject**	**S1**	**S2**	**S3**	**S4**	**S5**	**S6**	**S7**	**S8**	**S9**	**S10**	**S11**	**Mean**
Well-rested	2.52	2.48	2.31	2.04	2.24	2.75	1.94	2.80	1.94	2.01	2	2.34
Sleep-deprived	1.47	1.17	1.44	1.07	1.11	1.73	1.42	1.88	1.54	1.19	1.21	1.39
rDCL (%)	41.7	52.8	37.7	47.5	50.2	37.1	26.7	32.9	21	40.8	39.5	40.7

*Relative DCL (rDCL) indicates percentage of fatigue level by comparison to well-rested condition*.

**Figure 5:**
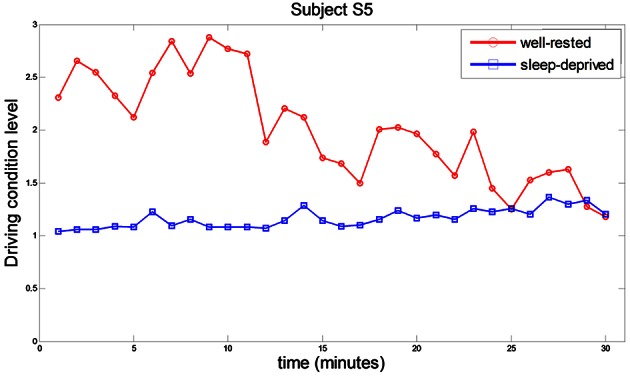
**Comparative driving condition level behavior in well-rested and sleep-deprived conditions while subject S5 was driving**. Each point represents the averaged DCL value during one minute of driving. Red circles and blue squares represent the DCL in the well-rested and sleep-deprived conditions, respectively.

To investigate the drivers' mental fatigue over time, each DCL value (per a minute) was estimated by extracting features of RPL (beta over alpha), HbO, and averaged HR. Figure 5 shows the DCL values of subject S5, in which the values decreased gradually over time in the well-rested condition, and remained consistent at approximately 1 in the sleep-deprived condition. Sleep-deprived subjects were quite fatigued already at the beginning of the driving task. From the questionnaire, we found a weak correlation (*r*^2^ = 0.42) between rDCL values in the sleep-deprived condition and subjects' reported degree of sleepiness (1: rarely sleepy to 5: very sleepy). The average scores for sleepiness over all subjects were 1.4 and 4.1 in the well-rested and sleep-deprived conditions, respectively, while the average hours of sleep reported were 7.36 and 0 h, respectively.

### Multimodal integration to determine neuro-physiological correlates

**Figure 6:**
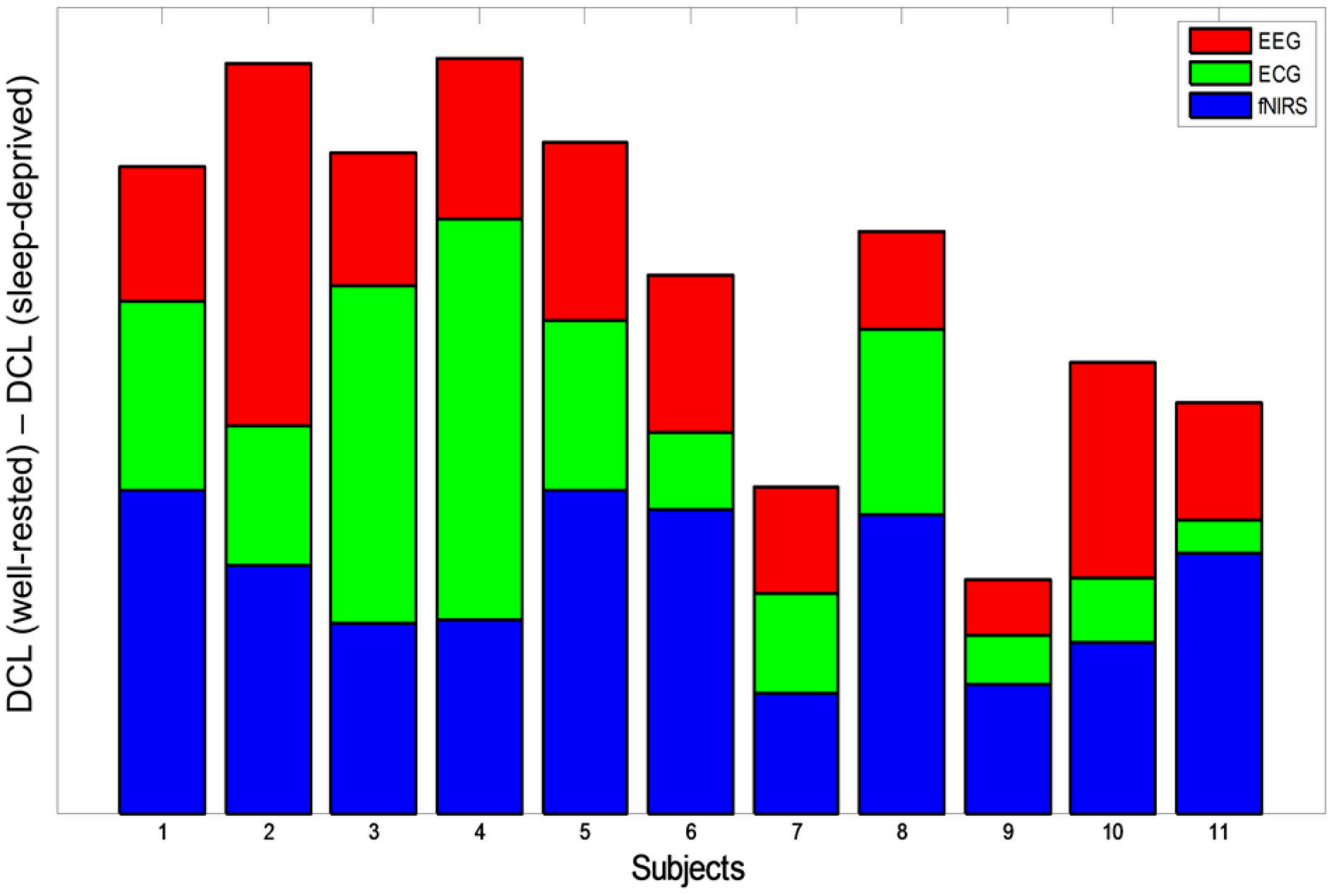
**DCL difference (subtraction of sleep-deprived from well-rested) for all subjects**. Each color represents modality-specific contribution to DCL difference (red: EEG, green: ECG, blue: fNIRS). Summation of the three colored bars indicates the synergistic effect of multimodal data on classification between the two driving conditions.

In this work, we recorded simultaneous EEG/ECG/EOG and fNIRS signals for multimodal analysis. Multimodal integration is an efficient technique that yields important insights into brain processes (Uludağ and Roebroeck, 2014). Even though the EOG signals did not differ statistically in this work, they were used to eliminate eye movement artifacts in the EEG data. On the other hand, EEG/ECG and fNIRS data yielded clear features that discriminated between the driving conditions, and each feature from the three different modalities differed significantly between the well-rested and sleep-deprived conditions. Based on these features, DCL (summation of these normalized factors) was proposed to determine neuro-physiological correlates of drivers' mental fatigue in a quantitative manner. As a result, we observed that DCL may offer a reasonable method to discriminate well between the two driving conditions. To illustrate the individual contribution of each modality to the differences in DCL between the two driving conditions, the modality-specific contributions are shown for all subjects in Figure 6. Accumulation of the three colored bars indicates DCL differences in the multimodal data (EEG+ECG+fNIRS), which represent the synergistic effect of these data. As shown in this figure, subjects S2 and S4 demonstrated the greatest differences in DCL, while the DCL of subjects S7 and S9 differed the least between the two conditions. Because of the unbiased combination, the averaged contributions of each modality (EEG, ECG, and fNIRS) to the DCL differences were quite similar (0.46, 0.45, and 0.46, respectively).

### Comparison of hybrid approaches using EEG/ECG and fNIRS

**Figure 7:**
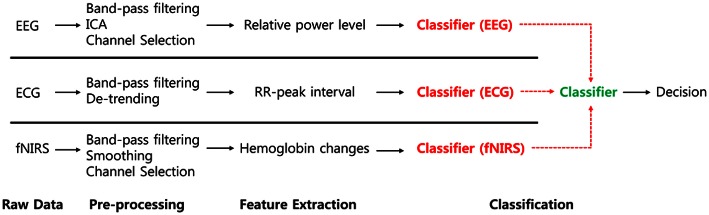
**Flow diagram for classifier combination**. Each classifier's output is regarded to second classifier's input.

To investigate the hybrid effect of the classification for the two different driving conditions, we compared various combinations of modalities with respect to the classifiers' outputs. For the combined classifiers in each modality, each classifier's outputs (EEG, ECG, and fNIRS) were regarded as features of the second classifier. Thereafter, the outputs of the second classifier represent the results of the combined classifiers. A flow diagram of this procedure is depicted in Figure 7.

Classification accuracies of single modalities (EEG, ECG, fNIRS) and all combinations of modalities (EEG+ECG, EEG+fNIRS, ECG+fNIRS, and EEG+ECG+fNIRS) at the classification level are summarized in Table 3. Most of the subjects (8 of 11) achieved improved performance in the combined EEG+ECG+fNIRS, and the average performance of this combination was greater than that of the others. A one-way ANOVA conducted on the seven different approaches indicated that there was a considerably significant difference [F_(6, 70)_ = 4.38, *p* = 0.0008 < 0.10]. Further, the EEG+ECG+fNIRS combinations differed significantly from the others (*p* < 0.05, Wilcoxon signed-rank test). Each level of significance is marked with asterisks in Table 3. Notably, subject S6 showed the greatest improvement (~30.5%) in the EEG+ECG+fNIRS combination compared to EEG only.

**Table 3:** **Classification accuracies in well-rested and sleep-deprived conditions**.

**Subject**	**S1**	**S2**	**S3**	**S4**	**S5**	**S6**	**S7**	**S8**	**S9**	**S10**	**S11**	**Mean**
^**^EEG	61.3	52.1	58.9	54.4	70.8	55.2	68.9	64.3	61.0	52.2	58.1	59.7
^**^ECG	79.4	72.0	52.8	76.3	60.5	52.3	63.9	72.2	61.2	58.8	59.7	64.5
^*^fNIRS	68.4	**84.5**	55.6	65.9	76.8	80.8	**72.9**	61.5	52.6	57.7	58.3	66.8
^**^EEG+ECG	76.2	60.0	**74.3**	68.1	72.9	68.9	64.3	73.5	71.4	60.4	69.5	69.0
^**^EEG+fNIRS	83.3	63.3	66.7	65.5	72.7	63.3	63.3	70.0	73.3	60.3	70.0	68.3
^**^ECG+fNIRS	82.8	64.8	63.3	73.3	73.3	73.3	60.0	76.7	63.3	56.7	73.3	69.2
EEG+ECG+fNIRS	**84.5**	73.7	71.8	**77.9**	**78.1**	**85.7**	67.0	**83.8**	**74.6**	**62.4**	**75.2**	**75.9**

The highest accuracies among seven approaches are displayed in bold. Asterisks indicate the level of signficance with the EEG+ECG+fNIRS combination (

** p < 0.01:

* *p < 0.05)*.

## Discussion

### EEG spectral changes and driving conditions

To date, most researchers have investigated driving fatigue using EEG changes, which are promising indicators of this phenomenon (Lal and Craig, 2001), and EEG has the advantages of being portable, noninvasive, inexpensive, and safe to measure during driving. With EEG recording, Lal and Craig (2002) found substantial increases in delta, theta, and alpha activity in the transition to fatigue, which was consistent with existing findings described in a review paper (Sahayadhas et al., 2012). Alpha activity is believed to be the most prominent indicator of driver fatigue. With this reasoning, Simon et al. (2011) verified alpha spindle activity based on a short-time Fourier transformation in real traffic conditions. Statistical analysis of these actual driving data revealed significant increases for all alpha spindle parameters, such as rate, duration, amplitude, and power, between the awake and drowsy states during 20 min of driving. Similarly, in the EEG recordings in this work, we observed a significant increase in alpha based on RPL. To reduce session and subject variability, a normalized alpha RPL was introduced and a significant alpha RPL difference was found in the centro-parietal region.

It is known that attention is also correlated with alpha power suppression. In our experiment, visual attention may be expected to be related closely to a simulated driving task. Such visual attention-related alpha power suppression may be observed normally in the occipital region, as reported in previous studies (Worden et al., 2000; Sauseng et al., 2005; Rihs et al., 2007). However, in our study, notable alpha suppression was observed in the centro-parietal region alone, which is consistent with results in previous studies of fatigue (Lal and Craig, 2001, 2002; Simon et al., 2011; Sahayadhas et al., 2012). For this reason, it is clear that a reduction in power in the alpha band was correlated with fatigue in this experiment. We observed beta RPL changes in the sleep-deprived condition, and beta power may be an additional indicator of mental fatigue. Tanaka et al. (2012) found that beta power densities decreased significantly after tiring cognitive tasks. They calculated EEG power spectra in each band and showed that beta waves decreased significantly in the fronto-central region with increased driving times. It also has been reported that beta rhythm is associated closely with increased alertness and arousal (Okogbaa et al., 1994), which is likely to be applicable to driving situations (Yeo et al., 2009; Yang et al., 2010; Zhao et al., 2012). In this work, we inferred that the lack of arousal and alertness caused by mental fatigue and sleep deprivation may result in a decreased beta rhythm during simulated driving.

Until now, most studies related to the detection of mental fatigue during driving have been experimental, and driving conditions have been divided according to the elapsed duration of driving. For example, data from the first 10 min have been considered to be the normal condition, while those from the last 10 min were specified as the fatigued condition (Li et al., 2009; Simon et al., 2011). Such an approach may not be appropriate, however, as some people may not develop fatigue even after 2–3 h of driving, especially professional drivers. Therefore, in order to discriminate between high- and low-risk conditions explicitly, each subject was both at high and low risk of fatigue before the driving tests, depending on how many hours they slept at night. Because sleep deprivation is well known to affect decision-making, attention, vigilance, human performance, and mental fatigue (Åkerstedt et al., 2004; Alhola and Polo-Kantola, 2007), it is appropriate to refer to sleep deprivation as analogous to fatigue. In addition, in our driving simulator, the steering wheel vibrated whenever the car crashed into a barrier to prevent drivers from actually falling asleep. Preventing the subjects from falling asleep may have suppressed activation in the delta and theta bands because these waves are associated closely with deep sleep (Maquet et al., 1997) and REM sleep (Jouvet, 1969), respectively.

### Observations of fatigue in the well-rested condition

**Figure 8:**
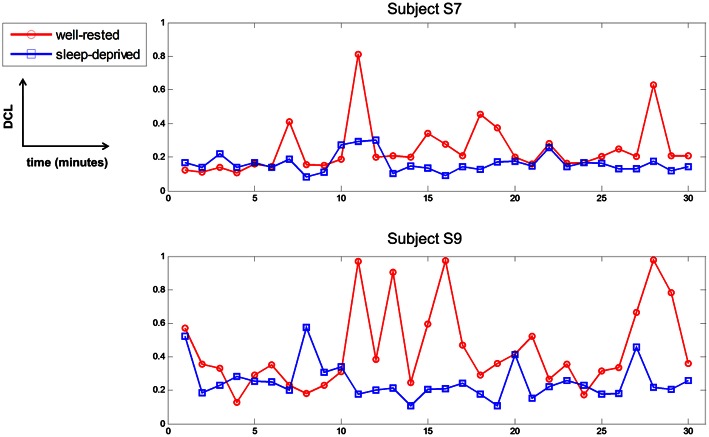
**Driving condition level of subject S7 and S9 in two conditions per 1 min over time**. Red-circle and blue-square represent the driving condition level in well-rested and sleep-deprived conditions, respectively.

Before the experiment, all subjects with well-rested condition were instructed to sleep over 7 h to ensure that they were mentally alert and physically refreshed. However, several subjects experienced fatigue in the driving task nonetheless, due to various internal or external environmental factors, even though they reported that they had slept well the previous night. Subjects S7 and S9 had the lowest DCL values in the well-rested condition, as shown in Table 2 and Figure 6. According to their questionnaires, these two subjects recorded a score of 2 in the sleepiness section (1: rarely sleepy to 5: very sleepy) prior to the experiment, yet they often nodded off during driving. Their behavior was recorded on the HD-Webcam, and showed clearly that they were drowsy. Furthermore, they reported scores of 4 and 5, respectively, on the sleepiness scale after the experiment. Figure 8 represents the DCLs for these two subjects over time. As shown, their DCLs in the well-rested condition were similar to those in the sleep-deprived condition. The DCLs fluctuated in the well-rested condition, as shown by repeated increases and decreases. Moreover, these two subjects achieved low classification accuracies in a single modality, as shown in Table 3, although their accuracy improved when measured with mixed features. Clearly, we believe that these two subjects were likely to have been fatigued despite their assignment to the well-rested condition. In this work, we used the HD-webcam only to monitor the subjects, and did not measure or analyze behavioral data. Analyzing subjects' behavior in real-time, such as head or eye movements, may offer supporting evidence that some subjects slept well but experienced mental fatigue nonetheless. We will collect such behavioral data in the online mental fatigue monitoring system, which is currently under investigation.

### Temporal mismatch between EEG and fNIRS

Recently, many studies have tried to combine EEG and fNIRS to improve classification accuracy or increase the degrees of freedom in BCI systems (Fazli et al., 2012; Khan et al., 2014; Putze et al., 2014; Koo et al., 2015; Yin et al., 2015). However, the fNIRS system measures hemodynamic change, which is a delayed response compared to neuronal electrical activity, and it also has a relatively low temporal resolution (<10 Hz), both of which are critical drawbacks in fNIRS measurements. Because of their low temporal resolution, it is sometimes difficult to combine fNIRS data with other brain imaging data. Nevertheless, one of the most significant merits of the fNIRS system is its ability to measure oxygen consumption related to blood flow in the brain, similar to that in functional magnetic resonance imaging (fMRI), and fNIRS has been nicknamed the portable fMRI for this reason.

Considering the advantages and drawbacks of the fNIRS system, we calculated the features during each 1-min period throughout the dataset in this work. Each minute in the 30 min of data yielded a DCL value, which was used to discriminate between the well-rested and sleep-deprived conditions. In addition, oxygen consumption in the prefrontal cortex may represent cognitive workload or fatigue (Ayaz et al., 2012, 2013; Harrison et al., 2014; McKendrick et al., 2014). Therefore, it is likely that fNIRS may be a significant indicator of mental fatigue, and we are sure that employing multimodal data is quite useful in monitoring mental fatigue. Also, the prefrontal cortex is related closely to mental workload (Mandrick et al., 2013a) and the performance of cognitive tasks (Mandrick et al., 2013b). For these reasons, we introduced the prefrontal fNIRS in this work. However, whole head fNIRS would be beneficial and will be considered in our future work.

### Limitations and future work

We proposed here an indicator of drivers' mental fatigue (Equation 4) that was able to discriminate the drivers' mental conditions well. To include equal contributions of EEG, ECG, and fNIRS features in the indicator, we normalized and summed all three. Further, all three were weighted equally to calculate the indicator, although this might not be an optimal method of quantification. Therefore, we calculated a new indicator using a weighting factor of $\left[DCL\;=a*norm\text{​}\left(\frac{beta\;RPL}{alpha\;RPL}\right)+b\;*\;norm\text{​}\left(HbO\right)+c\;*\;norm(HR)\right]$. Each weighting factor was defined between 0 and 1 with increments of 0.10; thus, we were able to search for the highest DCL difference in the two conditions (subtraction of sleep-deprived from well-rested). As a result, they had values comparable to those with equal weights and we observed no significant improvement. Even though we were able to apply elegant optimization methods, a somewhat better indicator may be achieved.

The primary reason to monitor drivers' mental fatigue is to prevent car accidents by providing drivers with a rapid and reliable alarm. To achieve this purpose, both making predictions before driving and monitoring a driver's condition in real-time are potential approaches. Our proposed rDCL (Equation 5) can predict a driver's fatigue prior to driving if training data can be obtained when the driver is in an alert state. Compared with DCL in the well-rested condition, the driver's condition prior to driving may be pre-checked by estimating the DCL. Although we used the entire dataset obtained during 30 min in the well-rested condition in this work, data of a shorter duration could be used for baseline. We are investigating the minimum duration needed to predict drivers' fatigue now. Another approach to accident prevention is to monitor drivers' fatigue in real-time.

We performed only an offline analysis in this work, and normalized DCL values were estimated each minute. For online monitoring, however, normalization of each modality's features is quite difficult to implement based on current methods available. One possible approach to solve the normalization issue is to record resting data before driving and use them as baseline data. Alternatively, an adaptive normalization method that updates feature values in real-time is a candidate. We are investigating the most appropriate normalization method for a subsequent online mental fatigue monitoring system.

In this work, we attempted to analyze multimodal data with simultaneous recordings of EEG/ECG/EOG and fNIRS. One of the advantages of multimodal signal integration is that each imaging method provides a physiologically and physically filtered view of one or more brain processes of interest. Thus far, the EEG-fMRI combination has been investigated widely, especially in epilepsy research, to help localize specific regions (Rosenkranz and Lemieux, 2010) by improving spatial and temporal resolution. Now, the EEG-fNIRS combination may be an alternative imaging method with merits that include low cost and simple implementation.

In this study, we custom-built an fNIRS that was already validated in previous study (Kim et al., 2015), and thus enabled us to design the experiment well. Although, a lengthy preparation time was required to attach the detectors and emitters, and test the quality of the light intensity for fNIRS measurement, this EEG-fNIRS integration may be quite beneficial in developing a monitoring system, as reported in the existing literature (Fazli et al., 2012; Wallois et al., 2012; Khan et al., 2014; Morioka et al., 2014; Putze et al., 2014; Koo et al., 2015; Yin et al., 2015). Another concern in analyzing multimodal data is how their differences (physical values, temporal resolution) are considered in an integrated frame. An in-depth investigation is needed to enhance the synergistic effect of multimodal data recording. Similarly, in this study, we were unable to guarantee that the EEG, ECG, and fNIRS, or their combined features, are related linearly to the fatigue level, although in the multimodal results, each modality influenced the fatigue level to some degree, as shown in Figure 8.

## Conclusions

The purpose of this study was to use simultaneous EEG/ECG/EOG and fNIRS recordings to determine neuro-physiological correlates that can be used to discriminate sleep deprivation-induced mental fatigue in drivers by comparison to those who are well-rested. To achieve our purpose, we introduced two driving conditions (well-rested and sleep-deprived), and were able to extract significant features from their EEG, ECG, and fNIRS data. However, no significant feature was found in the EOG due to high variability in the subjects' data. The features observed allowed us to determine the mental condition of each driver, and also yielded good discriminative results between two driving conditions. Further, we investigated the synergistic effects of multimodal data to compare the various combinations at the classification level with a single modality. In conclusion, our proposed combined approach of simultaneous EEG/ECG and fNIRS data may be a promising tool with which to monitor drivers' mental fatigue.

## Author contributions

SA, TN, HJ, JGK, and SCJ designed the experimental paradigm, and TN and JGK custom-built the fNIRS system. Data collection was conducted by SA, TN, and HJ, and analysis was performed by SA; the entire manuscript was written by SA and SCJ.

### Conflict of interest statement

The authors declare that the research was conducted in the absence of any commercial or financial relationships that could be construed as a potential conflict of interest.
